# Vitamin C status across the spectrum of chronic kidney disease and healthy controls: a cross-sectional study

**DOI:** 10.1016/j.ajcnut.2025.09.008

**Published:** 2025-09-08

**Authors:** Caecilia SE Doorenbos, Dian P Bolhuis, Karin JR Ipema, Ellen M Duym, Ralf Westerhuis, Mariken E Stegmann, Casper FM Franssen, Stephan JL Bakker, Antonio W Gomes-Neto, Gerjan Navis, Stefan P Berger, Akin Özyilmaz, TransplantLines Investigators, C Annema, H Blokzijl, FAJA Bodewes, MT de Boer, K Damman, MH de Borst, IJC Dielwart, A Diepstra, G Dijkstra, RM Douwes, MF Eisenga, ME Erasmus, CT Gan, AM Posthumus, E Hak, BG Hepkema, J Jonker, F Klont, TJ Knobbe, D Kremer, HGD Leuvenink, WS Lexmond, VE de Meijer, GJ Nieuwenhuis-Moeke, HGM Niesters, LJ van Pelt, RA Pol, AV Ranchor, JSF Sanders, MJ Siebelink, RJHJA Slart, JC Swarte, DJ Touw, MC van den Heuvel, C van Leer-Buter, M van Londen, Charlotte A te Velde-Keyzer, EAM Verschuuren, MJ Vos, RK Weersma

**Affiliations:** 1 University of Groningen, Division of Nephrology, Department of Internal Medicine, University Medical Center Groningen, Groningen, The Netherlands; 2 Department of Nephrology and Hypertension, University Medical Center Utrecht, Utrecht, The Netherlands; 3 University of Groningen, Department of Dietetics, University Medical Center Groningen, Groningen, The Netherlands; 4 Dialysis Center Groningen, Groningen, The Netherlands; 5 University of Groningen, Department of Primary and Long-term Care, University Medical Center Groningen, Groningen, The Netherlands

**Keywords:** chronic kidney disease, vitamin C deficiency, vitamin C intake, malnutrition, hemodialysis

## Abstract

**Background:**

Patients with chronic kidney disease (CKD) are at risk for vitamin C (VitC) deficiency.

**Objectives:**

We aimed to investigate VitC status and its determinants across the spectrum of CKD and healthy individuals.

**Methods:**

In this cross-sectional study, we measured plasma VitC concentrations (<10 *μ*mol/L = deficient, 10‒35 *μ*mol/L = inadequate) in 62 individuals on dialysis, 41 with CKD stage 4/5, 42 kidney transplant recipients (KTRs) (together: CKD population), and in 447 living kidney donors, and 385 healthy controls (together: healthy population). In the CKD population, we assessed VitC intake (<75 mg/d = inadequate) using 24-h dietary recalls. We measured VitC removal by hemodialysis. We investigated potential determinants of plasma VitC with linear regression in the CKD population and in the healthy population separately.

**Results:**

Median (Q1‒Q3) plasma VitC was highest in healthy controls [58 (43‒69) *μ*mol/L, 14% inadequate/deficient], followed by kidney donors [50 (36‒66) *μ*mol/L, 22% inadequate/deficient], KTR [37 (19‒46) *μ*mol/L, 48% inadequate/deficient], dialysis [33 (19‒47) *μ*mol/L, 58% inadequate/deficient], and CKD stage 4/5 [22 (14‒34) μmol/L, 80% inadequate/deficient]. Daily dietary VitC intake was similar in KTR [55.5 (33.5‒144.2) mg, 55% inadequate] and CKD stage 4/5 [53.0 (23.9‒142.0) mg, 56% inadequate], and higher in dialysis [113 (72‒209) mg, 26% inadequate] due to supplementation. VitC removal was 58 (27‒123) mg in conventional and 128 (97‒156) mg in nocturnal hemodialysis sessions. In the healthy population, estimated glomerular filtration rate was positively associated with plasma VitC [standardized β coefficient: 0.18 (0.08, 0.27), *P* < 0.001], independent of potential confounders.

**Conclusions:**

VitC inadequacy and deficiency are common in CKD. Inadequate intake and removal by dialysis may contribute. In healthy individuals estimated glomerular filtration rate was inversely associated with plasma VitC. Our findings highlight the importance of monitoring VitC status in CKD, and represent a potential target for intervention. Future research could evaluate the potential health effects of improving VitC status in CKD.

## Introduction

Chronic kidney disease (CKD) is a highly prevalent condition associated with reduced life expectancy and quality of life [1]. Patients with CKD are at risk for several micronutrient deficiencies, including vitamin C (VitC, ascorbic acid). VitC is a water-soluble antioxidant and cofactor for multiple enzymes, involved in many physiological processes, including immune function, iron absorption, and collagen synthesis [2,3], and may protect against anemia [4,5] and vascular disease [6,7]. Severe VitC deficiency can lead to a broad spectrum of health problems, including scurvy [3,[8], [9], [10]].

Under physiological conditions, excess VitC is filtered by the glomeruli and subsequently resorbed by proximal tubular transporters to maintain adequate plasma concentrations [3]. As estimated glomerular filtration rate (eGFR) declines in individuals with CKD, one might expect higher plasma concentrations due to reduced renal clearance of VitC, as is the case with its metabolite oxalate, for example [11]. Paradoxically, prior research suggests that patients with CKD exhibit lower plasma VitC concentrations compared to individuals with normal kidney function [[12], [13], [14]], which was associated with increased mortality [14]. Although underlying mechanisms are incompletely understood, potential explanations include lower intake [15] and malnutrition [16,17], removal through dialysis [18], and increased utilization of VitC due to increased oxidative stress [19]. Takahashi et al. [20] (2011) showed that worse eGFR was associated with lower plasma VitC concentrations in patients with CKD stage 3‒5 [20], raising the question whether there could also be a more direct relation between kidney function and VitC status. It is unknown whether kidney function in healthy individuals is also associated with plasma vitamin C concentrations.

We hypothesize that patients with CKD have poorer VitC status and intake compared to healthy individuals due to various contributing factors, including a lower VitC intake, potassium restriction, dialysis, and inflammation. Furthermore, if kidney function itself is associated with VitC status, we also expect that kidney donors (KDs) have lower VitC concentrations compared to healthy controls (HCs). In this study, we aimed to obtain a broader overview of VitC status, its determinants, and its relation to kidney function and CKD. Therefore, we comprehensively studied patients across the full spectrum of CKD, as well as healthy individuals.

## Methods

### Study population

All participants of this cross-sectional cohort study provided written informed consent. All study procedures were performed in accordance with the World Medical Association Declaration of Helsinki and the Declaration of Istanbul. This study has been approved by the local institutional review board (METc2014/033). The study population for this study comprises the CKD population and the healthy population, with data from the CKD population being obtained specifically for this study, and data from the healthy population being obtained from an existing cohort. Therefore, these study populations are described separately.

#### CKD population

Patients with various stages of CKD were included. Inclusion criteria were patients aged ≥18 y on conventional hemodialysis (CHD), on nocturnal in-center hemodialysis (NCHD), and on peritoneal dialysis (PD) from the Dialysis Center Groningen, together forming the dialysis group; patients with CKD stage 4/5 from the outpatient clinic of the University Medical Center Groningen (UMCG); and kidney transplant recipients (KTRs) from the UMCG. Exclusion criteria were the presence of active gastrointestinal or oncological diseases at the time of inclusion and use of VitC-containing supplements, except for routinely prescribed multivitamins in dialysis patients, which all patients in the dialysis group received. All patients in the CKD stage 4/5 group and in the dialysis group had regular contact with a dietician from the Dialysis Center Groningen or UMCG according to standard clinical practice. Other than that, study participants received no specific recommendations for VitC intake.

Patients in the CHD group were on a thrice weekly 2.5‒4-h dialysis schedule using double-needle dialysis (except 3 patients on single-needle dialysis), with effective blood and dialysate flows of 200‒400 mL/min and 500‒700 mL/min, respectively. Patients in the NCHD group received a 7.5-h single-needle dialysis treatment every other night, with effective blood and dialysate flows of 150‒200 mL/min and 300‒400 mL/min, respectively. All hemodialysis treatments were performed with low-flux polysulphone dialyzers and with low-molecular-weight heparin as anticoagulation. The dialysate temperature was 36‒36.5°C. Dialysate potassium concentration varied between 1 mmol/L and 3 mmol/L depending on the prevailing predialysis potassium concentration. Patients in the PD group received conventional, standard, potential of hydrogen (pH) 5.5, lactate-buffered StaySafe PD solutions (Fresenius Medical Care). All data from the CKD population were obtained during regular hospital/dialysis visits between 14 March, 2016 and 18 October, 2017. See the participant flow chart in Supplemental Figure 1.

#### Healthy population

For the healthy population, data were obtained from the ongoing, prospective TransplantLines Biobank and Cohort study from the UMCG in the Netherlands (clinicaltrials.gov identifier: NCT03272841). A detailed description of the study design, inclusion, and exclusion criteria has been described previously [[21], [62]]. The TransplantLines study protocol was approved by the local institutional review board (Medisch Ethische Toetsingsommissie 2014/077) and adheres to the UMCG Biobank Regulation. From June 2015, all solid organ transplantation patients and donors (aged ≥18 y) of the UMCG (The Netherlands) were invited to participate in the TransplantLines Biobank and Cohort Study. In the current study, we included all (potential) living KDs who were screened for kidney donation, or had already donated a kidney, and had available VitC data. Donors with postdonation VitC data available were included in the KD group, representing healthy individuals with slightly reduced kidney function. Donors with only predonation screening data (and no postdonation data) available were included in the HC group, representing healthy individuals with normal kidney function. In this manner, participants were assigned to only 1 group, with no overlap of participants between groups, as illustrated in the participant flow chart in Supplemental Figure 2. All data from the healthy population were gathered during regular hospital visits between February 2017 and October 2022.

### Assessment of VitC concentrations

For all study groups, blood samples were taken in EDTA-coated tubes, directly preceding the hospital visit, and in the dialysis group before the start of a hemodialysis session. Additionally, in the CHD and the NCHD group, dialysate samples were taken during dialysis. Blood samples were cooled on ice and taken to the laboratory for processing immediately after sampling. Blood plasma was deproteinized using a 5% trichloroacetic acid solution, and the samples were subsequently stored at ‒20^0^C. Dialysate samples were cooled on ice directly after sampling and were immediately stored at ‒20^0^C, with no additional processing. The difference in sample dilution—due to plasma samples being diluted with 5% trichloroacetic acid, whereas dialysate samples were not diluted—was accounted for when calculating the final VitC concentrations. The maximum storage time until final determination of VitC was 2 wk (stability studies in our laboratory showed that VitC concentrations were stable for ≥3 wk in samples stored at ‒20^0^C). For final determination of VitC in plasma and dialysate, VitC was transformed to dehydroascorbic acid and subsequently derivatized to 3-(1,2-hydroxyethyl) furo-[3,4-b] quinoxaline-1-one. Reversed-phase HPLC (Waters Alliance) with fluorescence detections (excitation 355 nm, emission 425 nm; Jasco FP2020) [22] was used for the final determination of VitC concentrations. The limit of quantification is 0.05 *μ*mol/L. The upper detection limit is 240 *μ*mol/L. In our study, there were no measurements below the limit of quantification or above the upper detection limit. Internal quality control for the laboratory assay was performed by analyzing 2 control samples of patient-like material in every analysis, exactly as the study samples: 1 with a high VitC concentration and 1 with a low VitC concentration. These control samples were stored at ‒80°C with a stability of 1 y. External quality control was performed several times per year, by analyzing a sample that was sent to us via the Royal College of Pathologists of Australasia, exactly as the rest of the samples, to check the correlation between different laboratories. Further details on the VitC measurements are available in Supplemental Text 1.

According to local reference values, plasma VitC concentrations were categorized as adequate (>35 *μ*mol/L), inadequate (>10‒35 *μ*mol/L), and deficient (≤10 *μ*mol/L).

### Assessment of dietary intake

In the CKD population (dialysis, CKD stage 4/5, and KTR), dietary intake, including intake of VitC, was assessed with a 24-h dietary recall, in which all dietary intake from the last 24 h was determined. The 24-h dietary recall was performed by a dietician, 1 d prior to the determination of plasma VitC. Subsequently, energy, protein, fat, carbohydrate, sodium, potassium, and VitC intake were calculated using a food measurement software program, based on the Dutch food composition table 2016 (Nederlands Voedingsstoffenbestand, EvryDiëtist) [23]. A 24-h VitC intake of <75 mg was considered an inadequate intake. VitC supplements were prescribed to all dialysis patients, according to routine care. Prescribed daily supplementary VitC intake in the dialysis group was calculated as the mean number of VitC-containing tablets per day, multiplied by 82.5 mg, the VitC content per tablet.

In the healthy population (KD and HC), data on dietary VitC intake and supplementation were available in a subset of the population. In this subset, intake was recorded through a semi-quantitative, self-administered food frequency questionnaire (FFQ) of 177 items, developed at Wageningen University and validated for the Dutch population [24], recording dietary intake during the past month in natural units (e.g., a slice of bread) or household measures (e.g., a teaspoon). The questionnaires were verified and converted into nutritional intake per day, including VitC intake, using the 2006 Dutch food composition table [25]. Use of VitC-containing supplements was retrieved from electronic patient records and was confirmed via telephone interviews with the patients. Information on the supplementation dose was not available.

Twenty-four hour recall reflects recent intake, whereas a FFQ reflects habitual intake. This may influence outcomes of measurements of VitC intake.

### Assessment of VitC clearance by hemodialysis

VitC clearance by CHD and NCHD was calculated based on VitC concentrations of afferent blood and efferent dialysate samples, which were taken thrice during a single hemodialysis session: at the beginning (T_1_), halfway through (T_2_), and at the end (T_3_) of the treatment. At T_1_, blood samples were taken immediately after the patient was connected to the dialyzer, whereas dialysate samples were taken 15 min after the start of the treatment. At T_2_ and T_3_, blood and dialysate samples were collected simultaneously. VitC clearance (Cl_vitc_) was calculated as follows:

$${Cl}_{vitc}=Q_{do}\times \left(C_{do}/C_{bi}\right)$$

Q_do_ denotes efferent dialysate flow, C_do_ and C_bi_ denote VitC concentrations in efferent dialysate and afferent blood sample, respectively. Efferent dialysate flow Q_do_ was calculated as follows, in which Q_di_ is the afferent dialysate flow:

$$Q_{do}=Q_{di}+ultrafiltration\;rate\;\left(+predilution\;flow\;rate\right)$$

Predilution flow rate was not used in any of the patients included. Therefore, efferent dialysate flow was determined using the afferent dialysate flow and the ultrafiltration rate. T_2_ and T_3_ were averaged to obtain the median VitC clearance; T_1_ was not used in this calculation since the blood and dialysate samples at this timepoint were not taken simultaneously [26]. The 3 VitC concentrations in the dialysate at T_1_, T_2_, and T_3_ were used to calculate total VitC removal (in micromole per session) during a dialysis treatment by calculating the AUC and multiplying this by the total dialysate volume per hour.

To estimate the VitC removal fraction from the total VitC in the extracellular volume (ECV) per dialysis session, we estimated ECV using the calculation of Moore et al. [27] (1963) for primary analyses. For sensitivity analyses, ECV was also calculated using equations of Brøchner-Mortensen et al. [28] (1982) based on body weight, and of Brøchner-Mortensen et al. [28] (1982) based on body surface area (BSA). BSA was calculated with the Dubois formula [*BSA (m*^*2*^*) = 0.007184 ∗ height (cm)*^*0.725*^ *∗ weight (kg)*^*0.4255*^]. For all different equations, ECV was estimated using predialysis weight, and was calculated both with and without the addition of the ultrafiltration volume to account for predialysis fluid retention. Total VitC in ECV was calculated by multiplying ECV by plasma VitC concentrations. VitC removal fraction from the ECV was calculated by dividing total VitC removal by total VitC in the ECV.

### Assessment of other variables

For all participants, age, sex, cardiovascular disease history, comorbidities, and medication use were obtained from medical records. Height, weight, systolic and diastolic blood pressure, and heart frequency were measured during the hospital/dialysis visits. BMI (in kg/m^2^) was calculated as weight (kilogram) divided by height (meter) squared. Hemoglobin, albumin, C-reactive protein (CRP), creatinine, parathyroid hormone, iron, ferritin, transferrin saturation, sodium, and potassium were determined using routine laboratory methods. Urine volume was measured from a 24-h urine collection prior to the hospital visit.

### Statistical analyses

SPSS Statistics version 23.0 (SPSS Corporation [29]) and R Studio version 4.4.1 (R Core Team [30]) were used for data analysis. Continuous data were reported as median and IQR (Q1‒Q3). Categorical data were reported as a number (valid percentage). Distributions of continuous variables were examined for normality using histograms and Q-Q plots. *P* values of <0.05 were considered statistically significant. Variables with >5 missing values were reported below the tables.

Comparisons of variables between study groups were performed with the Mann-Whitney U test. Correlations between variables were assessed using the Spearman Rank Correlation test to obtain correlation coefficients (*r*).

For a subset of the KD group, longitudinal data on VitC concentrations were available. Available timepoints in this subgroup consisted of the donation screening visit, the day of donation (both predonation), the early postdonation follow-up visit, and/or 5 y postdonation follow-up visit. Plasma VitC concentrations at these different time points were compared within individuals using a paired samples T-test.

Univariable and multivariable linear regression analyses were performed using the lm() function from base R. All linear regression analyses were performed as complete case analyses. In the CKD population, there were no cases with missing data on variables that were included in linear regression analyses. In the healthy population, cases with missing data were list-wise excluded, with the number of complete cases mentioned in the legend of the table. Prior to the analyses, skewed variables were log_2_-transformed to obtain a normal distribution. Outcomes were presented as standardized β [95% confidence interval (CI)], representing the increase in the dependent variable expressed in SDs with either a 1 SD increase for continuous independent variables, or a 1 unit increase for categorical independent variables.

In the CKD population (dialysis, CKD stage 4/5, and KTR combined), associations of potassium intake and energy intake with VitC intake, and of potassium intake with plasma potassium, were investigated with adjustment for study group and intake of energy, protein, fat, carbohydrate, and sodium in univariable and multivariable linear regression.

Furthermore, separately for the CKD population (dialysis, CKD stage 4/5, and KTR combined) and for the healthy population (KD and HC combined), we performed univariable and multivariable linear regression analyses to investigate theory-based potential determinants of plasma VitC concentrations: age, sex, BMI, Smoking, CRP, VitC intake (not in the healthy population), energy intake (not in the healthy population), eGFR (not in the CKD population) and creatinine (not in the CKD population). Due to collinearity, eGFR and creatinine were not included in multivariable analyses together.

Additionally, univariable and multivariable linear regression analyses were performed separately for the CKD population and the healthy population to investigate the association of medication use with plasma VitC concentrations. In multivariable analyses in the CKD population, we adjusted for age, sex, CRP, VitC intake, and study group, and in the healthy population, we adjusted for age, sex, CRP, BMI, smoking, eGFR, and study group. These adjustments were chosen based on outcomes of the previously mentioned linear regression analyses in which we assessed potential determinants of plasma VitC.

## Results

### Population characteristics

We included 41 patients on CHD, 11 patients on NCHD, and 10 patients on PD, together forming the dialysis group (*n* = 62); 41 patients with CKD stage 4/5; 42 KTR; 447 KD; and 385 HC. Population characteristics are presented in Table 1, showing the lowest median age in HC [57 (50‒66)] and the highest in dialysis [64 (47‒72)], and a larger proportion of females between KD and HC compared to the CKD groups. As expected, eGFR was highest in HC, followed by KD, KTR, and CKD stage 4/5, respectively. The study groups were mostly similar regarding length, weight, and BMI, although weight and BMI were slightly higher in the CKD stage 4/5 group. Blood pressure and prevalence of hypertension were higher in dialysis and CKD stage 4/5 compared to the other groups. Cardiovascular comorbidity was more prevalent in the dialysis group and the CKD stage 4/5 group compared to KTR. The prevalence of diabetes mellitus was lower in KD and HC compared to other groups. Medication use differed substantially across all groups, with lower medication use in KD and HC compared to the CKD groups. Characteristics for separate dialysis groups (CHD, NCHD, and PD) are shown in Supplemental Table 1.

**TABLE 1:** Population characteristics of study groups.

	Dialysis (*n* = 62)	CKD stage 4/5 (*n* = 41)	KTR (*n* = 42)	Kidney donor (*n* = 447)	Healthy control (*n* = 385)
Vitamin C status:					
Plasma concentration, *μ*mol/L	33 (19‒47) (range 3‒227)	22 (14‒34) (range 2‒204)	37 (19‒46) (range 3‒137)	50 (36‒66) (range 5‒149)	58 (43‒69) (range 2‒162)
Deficient (≤10 *μ*mol/L), *n* (%)	7 (11)	7 (17)	3 (7)	7 (2)	6 (2)
Inadequate (>10‒35 *μ*mol/L), *n* (%)	29 (47)	26 (63)	17 (41)	93 (21)	50 (13)
Adequate (>35 *μ*mol/L), *n* (%)	26 (42)	8 (20)	22 (52)	347 (78)	329 (86)
Characteristics:					
Female, *n* (%)	21 (34)	18 (44)	18 (43)	236 (53)	211 (55)
Age, years	64 (47‒72)	64 (60‒70)	59 (50‒67)	62 (54‒69)	57 (50‒66)
Height, cm	174 (167‒182)	172 (167‒181)	173 (165‒179)	173 (167‒180)	173 (167‒180)
Weight, kg	77 (67‒87)	88 (77‒93)	81 (70‒88)	82 (71‒93)	78 (69‒88)
BMI, kg/m^2^	25.3 (22.6‒28.6)	28.2 (25.3‒30.5)	26.3 (24.1‒30.1)	26.9 (24.5‒29.5)	26.1 (23.7‒28.4)
Systolic blood pressure, mmHg	143 (127‒160)	140 (130‒152)	130 (121‒144)	129 (119‒138)	125 (115‒134)
Diastolic blood pressure, mmHg	70 (60‒80)	79 (69‒87)	75 (70‒80)	75 (68‒82)	73 (67‒79)
eGFR mL/min/1.73 m^2^	N/A	10 (8‒14)	49 (41‒57)	63 (56‒71)	91 (80‒101)
Urine volume, L	0.4 (0.0‒9.0)	2.0 (1.5‒2.6)	2.3 (2.0‒2.9)	2.2 (1.6‒2.7)	2.3 (1.7‒2.8)
Time since kidney donation, months	N/A	N/A	N/A	62 (60‒119) (range 1‒184)	N/A
Comorbidities:					
Cardiovascular disease history, *n* (%)	31 (50)	23 (56)	13 (31)	missing	missing
Hypertension, *n* (%)	37 (60)	26 (63)	21 (50)	137 (31)	96 (25)
Diabetes mellitus, *n* (%)	18 (29)	13 (32)	10 (24)	1 (0)	1 (0)
Medication:					
Diuretics, *n* (%)	31 (50)	23 (56)	12 (29)	21 (5)	20 (5)
RAAS inhibition, *n* (%)	14 (23)	20 (49)	15 (36)	26 (6)	17 (5)
β-blocker, *n* (%)	36 (58)	25 (61)	27 (64)	21 (5)	11 (3)
Calcium antagonist, *n* (%)	25 (40)	23 (56)	16 (38)	16 (4)	15 (4)
Cholesterol-lowering agent, *n* (%)	25 (40)	25 (61)	24 (57)	27 (6)	27 (7)
Oral antidiabetics, *n* (%)	1 (2)	1 (2)	8 (19)	0 (0)	1 (0)
Insulin, *n* (%)	12 (19)	9 (22)	5 (12)	0 (0)	0 (0)
Corticosteroids, *n* (%)	14 (23)	4 (10)	42 (100)	1 (0)	1 (0)
Calcineurin inhibitor, *n* (%)	1 (2)	1 (3)	26 (62)	0 (0)	0 (0)
Acetylsalicylic acid, *n* (%)	30 (48)	14 (34)	11 (26)	12 (3)	12 (3)
Proton pump inhibitor, *n* (%)	33 (53)	17 (42)	35 (83)	28 (7)	38 (10)
H_2_-antagonist, *n* (%)	12 (19)	11 (27)	4 (10)	1 (0)	1 (0)
Iron suppletion, *n* (%)	49 (79)	20 (49)	4 (10)	0 (0)	1 (0)
ESA, *n* (%)	54 (87)	15 (37)	1 (2)	0 (0)	0 (0)
Laboratory measurements:					
Hemoglobin, mmol/L	7.0 (6.4‒7.5)	7.1 (6.4‒7.8)	8.3 (7.7‒9.2)	9.0 (8.5‒9.6)	9.0 (8.5‒9.6)
Creatinine, mmol/L	822 (680‒983)	411 (339‒522)	127 (107‒148)	99 (88‒115)	74 (66‒84)
Albumin, g/L	40 (37‒43)	42 (39‒44)	43 (41‒45)	44 (43‒46)	44 (43‒46)
CRP, mg/L	4.1 (1.6‒9.0)	4.2 (1.8‒9.0)	2.6 (0.9‒5.0)	1.4 (0.7‒2.7)	1.1 (0.5‒2.1)
Iron, *μ*mol/L	10.0 (7.6‒14.6)	11.9 (10.1‒16.5)	14.1 (11.5‒17.1)	15.5 (12.7‒19.2)	16.8 (13.3‒20.0)
Ferritin, *μ*g/L	366 (186‒517)	118 (86‒285)	87 (30‒181)	128 (74‒212)	124 (71‒215)
Transferrin saturation, %	23.0 (15.5‒30.0)	21.5 (17‒29.5)	21.5 (16.8‒29.3)	25 (21‒31)	27 (21‒33)
PTH, pmol/L	26.5 (12.0‒45.0)	22.1 (14.1‒37.7)	9.2 (7.0‒13.8)	5.5 (4.4‒6.9)	4.8 (3.8‒5.8)
Plasma sodium, mmol/L	138 (136‒139)	140 (139‒142)	140 (138‒143)	142 (140‒143)	140 (139‒141)
Plasma potassium, mmol/L	5.0 (4.5‒5.4)	4.8 (4.4‒5.2)	4.1 (3.6‒4.4)	4.1 (3.9‒4.3)	3.9 (3.8‒4.1)

Missing values: systolic blood pressure 8 in KD; urine volume 19 in CKD stage 4/5, 18 in KTR; RAAS inhibition 18 in HC; Proton pump inhibition 25 in KD, 18 in HC; Medication, 25 in KD, 18 in HC; hemoglobin 23 in KD, 12 in HC; CRP 9 in dialysis, 6 in CKD stage 4/5; ferritin 7 in CKD stage 4/5; iron 14 in dialysis, 8 in CKD stage 4/5; transferrin 8 in CKD stage 4/5; transferrin saturation 33 in dialysis, 9 in CKD stage 4/5; PTH 7 in CKD stage 4/5.

Abbreviations: BMI, body mass index; CKD, chronic kidney disease; CRP, C-reactive protein; eGFR, estimated glomerular filtration rate; ESA, erythropoiesis-stimulating agent; HC, healthy control; KD, kidney donor; KTR, kidney transplant recipient; N/A, not applicable; PTH, parathyroid hormone; RAAS, renin-angiotensin-aldosterone system.

### Plasma VitC status

**FIGURE 1:**
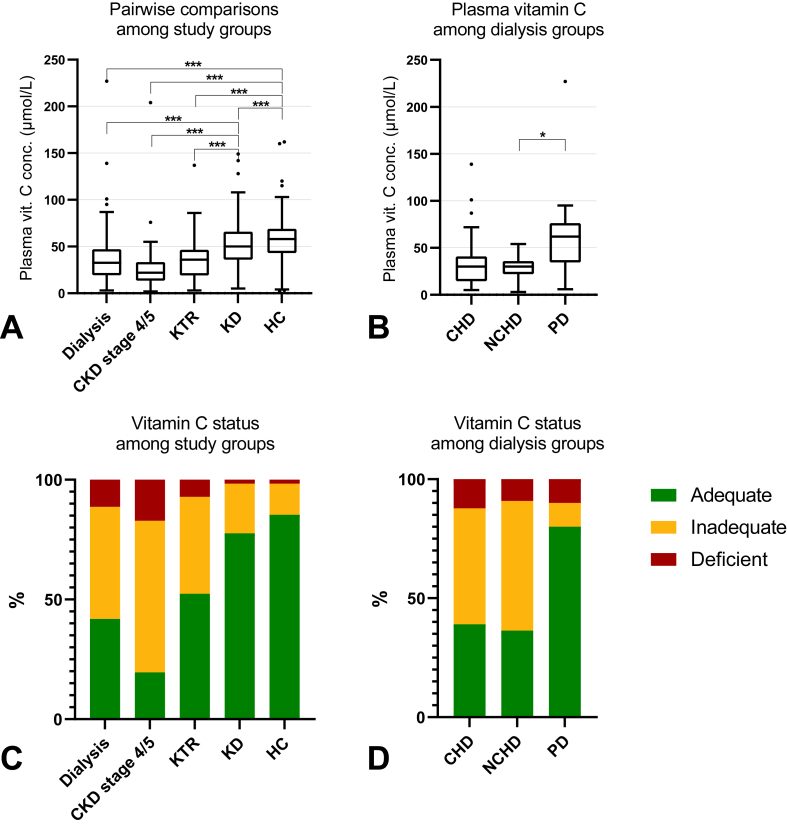
(A) Pairwise comparisons of vitamin C (vitC) concentrations with the Mann-Whitney U test with Bonferroni correction of plasma vitC concentrations across study groups; (B) Pairwise comparisons of vitC concentrations with the Mann-Whitney U test with Bonferroni correction of plasma vitC concentrations across separate dialysis groups; (C) VitC status across study groups; (D) VitC status across separate dialysis groups. ∗*P* < 0.05, ∗∗∗*P* < 0.001. CHD, conventional hemodialysis; CKD, chronic kidney disease; conc, concentration; HC, healthy controls; KD, kidney donor; KTR, kidney transplant recipient; NCHD, nocturnal in-center hemodialysis; PD, peritoneal dialysis.

Median plasma VitC concentrations were highest in HC [58 (43‒69) *μ*mol/L, 15% inadequate/deficient], followed by KD [50 (36‒66) *μ*mol/L, 22% inadequate/deficient], KTR [37 (19‒46) *μ*mol/L, 48% inadequate/deficient], dialysis [33 (19‒47) *μ*mol/L, 58% inadequate/deficient], and CKD stage 4/5 [22 (14‒34) *μ*mol/L, 81% inadequate/deficient], respectively (Table 1). VitC concentrations were significantly higher in KD and HC compared to the other study groups (Figure 1A). Within the dialysis group, we observed a striking difference between patients on PD and patients on hemodialysis: plasma VitC concentrations were highest in the PD group [62 (35‒76) *μ*mol/L, 70% adequate], followed by the CHD group [30 (15‒41) *μ*mol/L, 39% adequate] and the NCHD group [30 (22‒36) μmol/L, 27% adequate] (Supplemental Table 1). VitC concentrations were significantly higher in PD compared to NCHD (Figure 1B). VitC status distributions per group are shown in Figure 1C and D.

**FIGURE 2:**
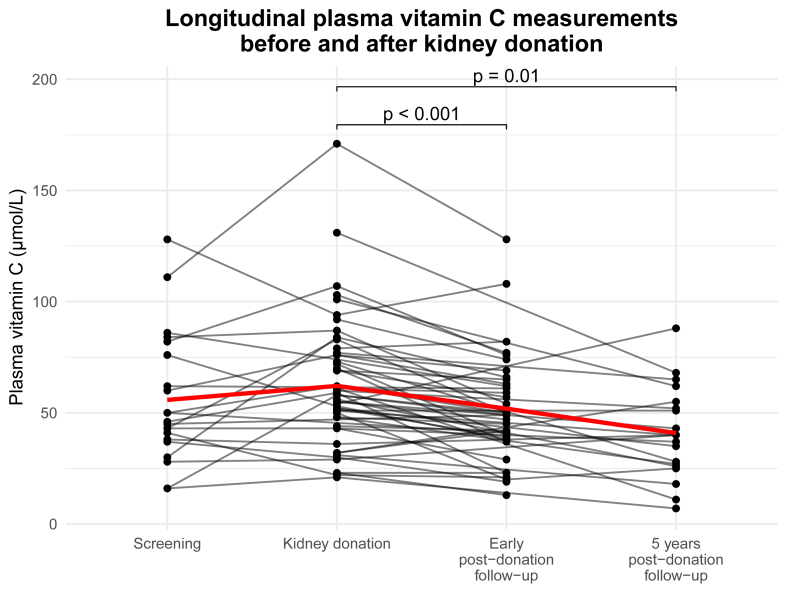
Plasma vitamin C concentrations at predonation screening [5.3 (2.8‒8.8) mo predonation, *n* = 20], at the day of donation (predonation, *n* = 51), at the early postdonation follow-up visit [1.7 (1.6‒2.1) mo postdonation, *n* = 35], and at the 5 y postdonation follow-up visit [60.2 (59.8‒60.8) mo postdonation, *n* = 20]. Vitamin C concentrations at different timepoints were compared with a paired T-test. The red line represents the mean vitamin C concentration across all timepoints.

In 55 KD, plasma VitC was measured before and after kidney donation. Concentrations were 46 (38–76) *μ*mol/L at the predonation screening [5.3 (2.8–8.8) mo before donation, *n* = 20], 58 (46–76) *μ*mol/L on the day of donation (presurgery, *n* = 51), 49 (39–64) *μ*mol/L at early follow-up [1.7 (1.6–2.1) mo postdonation, *n* = 35], and 40 (27–53) *μ*mol/L at 5-y follow-up [60.2 (59.8–60.8) mo postdonation, *n* = 20]. Levels declined significantly from the day of donation to early follow-up (*P* < 0.001) and to 5-y follow-up (*P* = 0.01) (Figure 2).

### Dietary intake

In the CKD population, median daily dietary VitC intake derived from 24-h dietary recalls was similar in dialysis [50.0 (25.0‒98.0) mg, 69% inadequate], CKD stage 4/5 [53.0 (23.9‒142.0) mg, 56% inadequate], and KTR [55.5 (33.5‒144.2), 55% inadequate), respectively (Table 2). As a result of prescribed vitamin suppletion in the dialysis group, daily total VitC intake was highest in the dialysis group [113 (72‒209) mg]. Energy consumption was relatively low in all groups. Macronutrient consumption was generally comparable between groups, with slightly lower carbohydrate intake in dialysis, slightly lower fat intake in CKD stage 4/5, and slightly higher protein intake in KTR. Sodium intake was higher in KTR. Potassium intake was lowest in dialysis, followed by KTR and CKD stage 4/5, respectively, despite the fact that the CKD stage 4/5 group had the highest rate of dietary potassium restriction. In separate dialysis groups, the CHD group consumed less VitC, energy, protein, fat, sodium, and potassium, and more carbohydrates compared to NCHD and PD (Supplemental Table 2). Prescribed VitC suppletion was highest in NCHD, followed by PD and CHD.

**TABLE 2:** Dietary intake of chronic kidney disease groups.

	Dialysis (*n* = 62)	CKD stage 4/5 (*n* = 41)	KTR (*n* = 42)
Dietary vitamin C intake, mg	50 (25‒98)	53 (24‒142)	56 (34‒144)
Dietary vitamin C intake inadequacy (≤75 mg/d), *n* (%)	43 (69)	23 (56)	23 (55)
Prescribed vitamin C supplementation, mg	35 (35‒83)	0 (0‒0)	0 (0‒0)
Total vitamin C intake (dietary + supplementary), mg	113 (72‒209)	53 (24‒142)	56 (34‒144)
Total vitamin C intake inadequacy (≤75 mg/d), *n* (%)	16 (26)	23 (56)	23 (55)
Energy intake, kcal	1567 (1241‒1984)	1556 (1286‒2081)	1788 (1509‒2053)
Carbohydrate intake, g	169 (137‒207)	206 (143‒244)	205 (164‒244)
Protein intake, g	65 (52‒84)	61.9 (51.7‒77.2)	71.4 (60.3‒79.8)
Fat intake, g	67 (46‒88)	58.4 (39.6‒80.0)	66.7 (50.4‒78.3)
Sodium intake, mg	1675 (1077‒2082)	1561 (1127‒2209)	2109 (1538‒2474)
Potassium intake, mg	2227 (1584‒2639)	2823 (1832‒3660)	2756 (2376‒3519)
Potassium restriction, *n* (%)	28 (45)	23 (56)	2 (5)

Missing values: none.

Abbreviations: CKD, chronic kidney disease; KTR, kidney transplant recipients.

In the CKD population (dialysis, CKD stage 4/5, and KTR combined), multivariable linear regression analyses showed that potassium intake was positively associated with VitC intake [st. β: 95% CI: 0.60 (0.41, 0.80), *P* < 0.001], and that potassium intake was not associated with plasma potassium, independently of study group and of energy, protein, fat, carbohydrate, and sodium intake. Energy intake was not associated with VitC intake.

In the healthy population, 11 KD had available data on daily dietary VitC intake derived from FFQ [103.9 (81.3‒112.3) mg], and 127 HC had available FFQ data on daily VitC intake [105.9 (72.0‒144.7) mg]. In 15 KD with available data on VitC supplementation, 2 (13%) used supplementation. In 188 HC with available data on VitC supplementation, 43 (23%) used supplementation, and had higher plasma VitC concentrations [66 (52‒79) *μ*mol/L] compared to HC not using VitC supplementation [56 (42‒66) *μ*mol/L] (*P* < 0.001).

### VitC clearance by hemodialysis

**FIGURE 3:**
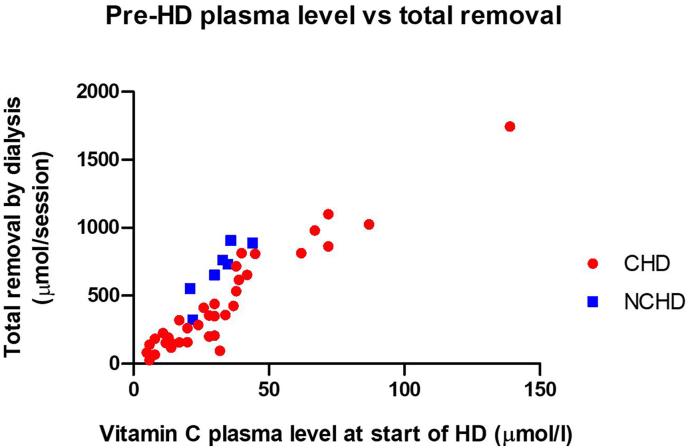
Relationship between predialysis plasma vitamin C concentration and total vitamin C removal during a dialysis treatment in CHD and NCHD. CHD, conventional hemodialysis; HD, hemodialysis; NCHD, nocturnal in-center hemodialysis.

Forty patients on CHD and 7 patients on NCHD had complete data on VitC clearance (Supplemental Table 3). Median VitC clearance in the CHD group was 87.1 (64.1‒106.6) mL/min, compared to 68.4 (63.0‒77.6) mL/min in the NCHD group (*P* = 0.08). Total VitC removal during dialysis was lower in the CHD group with a loss of 58 (27‒127) mg, compared to the NCHD group with a loss of 128 (97‒156) mg (*P* = 0.04). The fraction of the total VitC present in the ECV before dialysis that is removed by dialysis was 0.80 (0.57‒0.92) in CHD, compared to 1.26 (1.12‒1.31) in NCHD (*P* < 0.001) in primary analysis. In sensitivity analyses, median removal fractions ranged from 0.70 to 1.01 in CHD and from 1.04 to 1.60 in NCHD when using other calculations for ECV. Total VitC removal during dialysis treatment was positively correlated with the predialysis VitC plasma concentration in the CHD group (*r* = 0.906, *P* < 0.001) as well as in the NCHD group (*r* = 0.893, *P* = 0.007) (Figure 3).

### Determinants of plasma VitC concentration

In the CKD population (dialysis, CKD stage 4/5, and KTR combined), VitC intake was positively associated with log_2_-transformed plasma VitC concentration [st. β: 95% CI: 0.36 (0.20, 0.51), *P* < 0.001]. CRP was negatively associated with plasma VitC concentration [st. β: 95% CI: ‒0.21 (‒0.39, ‒0.04), *P* = 0.02]. In multivariable analysis, adjusting for age, sex, BMI, smoking, energy intake, and study group, these associations remained (Table 3). Furthermore, use of acetylsalicylic acid was negatively associated with plasma VitC in univariable analyses [st. β: 95% CI: ‒0.42 (‒0.76, ‒0.09), *P* = 0.01]. This association became stronger after adjustment for age, sex, CRP, and VitC intake (Supplemental Table 4).

**TABLE 3:** Results of univariable and multivariable linear regression analyses in the chronic kidney disease (CKD) population (dialysis group, the CKD stage 4/5 group, and the kidney transplant recipients group combined), with log_2_-transformed plasma vitamin C concentrations as the dependent variable. In the multivariable analysis, all potential determinants were included in the model together with additional adjustment for study group.

	Univariable	*P* value	Multivariable	*P* value
	St. β (95% CI)		St. β (95% CI)	
Age	‒0.00 (‒0.17, 0.16)	0.99	0.11 (‒0.07, 0.29)	0.24
Female	0.23 (‒0.11, 0.56)	0.18	0.23 (‒0.11, 0.57)	0.19
BMI	‒0.03 (‒0.20, 0.13)	0.69	0.02 (‒0.14, 0.18)	0.80
Smoking	0.00 (‒0.41, 0.41)	0.99	0.10 (‒0.34, 0.54)	0.65
CRP^1^	‒0.21 (‒0.39, -0.04)	0.02	‒0.23 (‒0.40, ‒0.06)	0.009
Vitamin C intake^1^	0.36 (0.20, 0.51)	<0.001	0.39 (0.20, 0.58)	<0.001
Energy intake	0.04 (‒0.12, 0.21)	0.63	‒0.13 (‒0.31, 0.05)	0.17

Missing values: none.

Abbreviations: BMI, body mass index; CI, confidence interval; CRP, C-reactive protein; st. β, standardized β coefficient.

1 Log_2_-transformed.

In the healthy population (KD and HC combined), female [st. β: 95% CI: 0.72 (0.59, 0.85), *P* < 0.001], and eGFR [st. β: 95% CI: 0.12 (0.05, 0.19), *P* < 0.001] were positively associated with plasma VitC concentrations, whereas BMI [st. β: 95% CI: ‒0.20 (‒0.27, ‒0.14), *P* < 0.001], smoking [st. β: 95% CI: ‒0.53 (‒0.70, ‒0.35), *P* < 0.001], plasma creatinine [st. β: 95% CI: ‒0.31 (‒0.37, ‒0.25), *P* < 0.001] and CRP [st. β: 95% CI: ‒0.08 (‒0.15, ‒0.01), *P* = 0.02] were negatively associated. In multivariable analysis, adjusting for each other and additionally for study group, these associations remained (Table 4), except for the association of CRP with plasma VitC. Importantly, in a subset of the healthy population with available data on intake (derived from FFQ) and supplementation, the association of eGFR with plasma VitC remained after adjustment for dietary VitC intake and VitC supplementation (data available in 133 participants). There were no statistically significant associations of medication use with plasma VitC, but use of proton pump inhibitors tended to be inversely associated with plasma VitC [st. β: 95% CI: ‒0.24 (‒0.50, 0.01), *P* = 0.06], also when adjusting for age, sex, CRP, BMI, and smoking (Supplemental Table 5).

**TABLE 4:** Results of univariable and multivariable linear regression analyses in the healthy population (kidney donor group and the healthy control group combined), with plasma vitamin C concentration as the dependent variable. In the multivariable analysis, all potential determinants were included in the model together with additional adjustment for the study group. Estimated glomerular filtration rate and creatinine were not included in the model together due to collinearity; therefore, separate multivariate models were performed.

	Univariable	*P* value	Multivariable	*P* value	St. β (95% CI)	*P* value
	St. β (95% CI)		St. β (95% CI)			
Age	0.06 (‒0.01, 0.13)	0.09	0.06 (‒0.01, 0.13)	0.08	0.01 (‒0.05, 0.08)	0.69
Female sex	0.72 (0.59, 0.85)	<0.001	0.57 (0.46, 0.69)	<0.001	0.39 (0.24, 0.54)	<0.001
BMI	‒0.20 (‒0.27, ‒0.14)	<0.001	‒0.19 (‒0.25, ‒0.13)	<0.001	‒0.19 (‒0.25, ‒0.13)	<0.001
Smoking	‒0.53 (‒0.70, ‒0.35)	<0.001	‒0.51 (‒0.68, ‒0.34)	<0.001	‒0.50 (‒0.67, ‒0.33)	<0.001
eGFR	0.12 (0.05, 0.19)	<0.001	0.18 (0.08, 0.27)	<0.001		
Creatinine	‒0.31 (‒0.37, ‒0.25)	<0.001			‒0.18 (‒0.27, ‒0.09)	<0.001
CRP^1^	‒0.08 (‒0.15, ‒0.01)	0.02	0.02 (‒0.05, 0.08)	0.63	0.02 (‒0.05, 0.08)	0.61

Missing values: smoking 39 in KD, 45 in HC (746 complete cases in multivariable analysis).

Abbreviations: BMI, body mass index; CI, confidence interval; CRP, C-reactive protein; eGFR, estimated glomerular filtration rate; HC, healthy control; KD, kidney donor; st. β, standardized β coefficient.

1 Log_2_-transformed.

## Discussion

In this study, we comprehensively described VitC status across the whole spectrum of CKD and HCs, showing that worse kidney function is associated with lower plasma VitC concentrations, even in healthy individuals. This novel finding is underscored by decreasing VitC concentrations following kidney donation. Strikingly, VitC inadequacy (10‒35 *μ*mol/L) and deficiency (≤10 *μ*mol/L) were present in more than half of the CKD population.

Study groups with worse kidney function had lower VitC concentrations. An exception was the dialysis group, with higher VitC concentrations compared to CKD stage 4/5, likely due to routinely prescribed VitC supplementation in dialysis. A previous study also found lower VitC concentrations in KTR compared to HCs [13]. Between CKD groups, VitC deficiency rates were 7‒17%, and deficiency rates in KD (2%) and HC (2%) were similar compared to <3% in large European general population studies. Notably, in more than half of the CKD population, VitC concentrations were below the adequate threshold.

These observations suggest kidney function could potentially be related to VitC status, which was further supported by a positive association between eGFR and plasma VitC in healthy individuals (KD and HC), independently of VitC intake and supplementation. Furthermore, plasma VitC decreased after kidney donation. Although this may partially be affected by postoperative oxidative stress in early stages after donation [31], we also observed decreased VitC concentrations in stable KDs at 5 y postdonation. Previously, a positive association of eGFR with plasma VitC was observed in CKD [20]. To our knowledge, we are the first to describe this association in healthy individuals, and in response to a circumscript reduction in kidney function.

A potential explanation for the association of kidney function with VitC could be excessive urinary VitC excretion despite suboptimal plasma concentrations. This may potentially happen due to dysfunctional resorption in the proximal tubule, or due to exceeding the resorptive capacity if VitC filtration per nephron increases in response to a decreased number of functional nephrons. This potential “renal leak” could be investigated by orally giving individuals with CKD and HCs isotopically-labeled VitC (using a carbon-13 or deuterium label), and subsequently measuring total urinary excretion of the administered dose.

Another potential explanation could be a reaction of VitC with benzoic acid, which is normally excreted by the kidney, and accumulates in the body when kidney function declines [32,33]. In foods and beverages, benzoic acid can react with VitC, forming benzene [34]. If this reaction also takes place in human body with elevated concentrations of benzoic acid, this reaction could potentially deplete plasma VitC.

An important finding of our study was the poor and often inadequate VitC intake between the CKD groups. The generally low energy intake reflects the well-documented loss of appetite [15] and malnutrition [16,17] associated with CKD, and may, although not apparent from our data, contribute to low VitC intake. As potassium intake was strongly associated with VitC intake, potassium restriction may also contribute to low VitC intake, since many foods containing VitC (e.g., fruits and vegetables) are high in potassium [3]. Meanwhile, widely implemented dietary potassium restriction may not be fully justified in the majority of patients with CKD [35]. In our study, potassium intake was not associated with plasma potassium, and when present in existing literature, this association is often weak [36,37].

Routine VitC supplementation in patients with CKD stage 4/5 and KTR, and increasing the dose in dialysis, could be considered as a potential, simple strategy to improve VitC intake. However, dietary improvement should remain the priority. Higher fruit and vegetable intake not only contributes to the intake of VitC, but also of other nutrients, yielding the broader benefits of a nutrient-dense diet. Potassium restriction should be carefully and individually considered, and plant-based potassium should be consumed over processed foods with potassium-based additives. Potassium binders could be considered to allow liberal fruit and vegetable intake. After kidney transplantation, the withdrawal of any potassium restriction should be emphasized.

In patients on dialysis, VitC is lost to the dialysate [18,38,39]. We demonstrated higher VitC removal during NCHD sessions compared to CHD. The fraction of estimated circulating VitC (in ECV predialysis) that was removed by dialysis was high, and often >1, indicating that VitC is also lost from the intracellular space. Plasma VitC was not different between CHD and NCHD, likely due to higher VitC supplementation in patients on NCHD. The hemodialysis patients had a worse VitC status compared to PD, similar to results of a previous study [39], probably due to a more efficient removal of water-soluble substances in hemodialysis compared to PD [40,41].

Besides kidney function, diet, and dialysis, other factors may influence VitC status. Patients with CKD exhibit increased inflammation and oxidative stress [19,42], causing a higher antioxidant demand. In the CKD population, higher CRP was associated with lower plasma VitC. In the healthy population, smoking was associated with lower plasma VitC, likely due to smoking-induced oxidative stress [43,44], although there may also be confounding by lifestyle or socio-economic status. Next to inflammation and oxidative stress, there may be other biochemical mechanisms that contribute to increased VitC utilization. Suboptimal health is associated with lower VitC status from a myriad of mechanisms, some of which are not well-defined. The lower VitC in the CKD population, and potentially also in the KD group, may be a general indicator that the health of these patients is to some extent compromised. There is substantial evidence that poor health in different types of chronic disease is associated with lower plasma VitC, with the decreased plasma VitC being a direct outcome of the illness [[45], [46], [47], [48], [49], [50]]. Some medications may also potentially affect plasma VitC, including acetylsalicylic acid, loop diuretics, proton pump inhibitors, and corticosteroids [[51], [52], [53], [54], [55]]. In our study, we indeed observed negative associations of acetylsalicylic acid and proton pump inhibitors. Consistent with literature [39], female sex was associated with higher plasma VitC, possibly due to a higher intake [[56], [57], [58]].

Although VitC deficiency is often considered a problem of the past, our findings suggest a reason for concern in the CKD population. Even in the HCs 14% had inadequate VitC concentrations, suggesting there might be a broader issue. Our findings may be relevant for CKD and for general health, as inadequate plasma VitC is associated with increased risk of morbidity and mortality [59]. Our adequacy threshold (≥35 *μ*mol/L) may even be conservative, as many studies recommend VitC concentrations of ≥50 *μ*mol/L for protection against cardiovascular and cancer-related morbidity and mortality [10,[59], [60], [61]].

As we investigated a predominantly Caucasian study cohort, our findings should be validated in other (patient) cohorts with different ethnic backgrounds. Furthermore, it would be valuable to conduct prospective, longitudinal studies measuring plasma VitC concentrations in patients with progressive CKD, and in living KDs before and after donation.

Limitations of the study include the single 24-h dietary recalls, as repeated 24-h dietary recalls might offer more reliable measurements. Under- or overreporting of food intake cannot be ruled out, and uncertainty in nutrient intake estimation is a general limitation of dietary assessment methods. Supplementation data, however, were very reliable in the CKD population. Only limited data on dietary intake were available in the healthy population, and these data were derived from FFQ as opposed to 24-h dietary recalls. This should be taken into account when comparing VitC intake between these groups. Due to the observational nature of the study, we cannot prove causality, and there can be residual confounding.

In conclusion, we demonstrate a high prevalence of VitC inadequacy and deficiency in CKD. Inadequate intake of VitC and removal during hemodialysis may contribute. Notably, poorer kidney function is associated with lower plasma VitC concentrations, even in healthy individuals. This novel finding emphasizes the need for future research to explore potential causal mechanisms. Our findings highlight the importance of monitoring VitC status in individuals with impaired kidney function, and suggest opportunities for targeted interventions to improve VitC status.

## Author contributions

The authors’ responsibilities were as follows– CSED: formal analysis, writing – original draft; DPB: analysis and writing; KJRI: conceptualization, methodology, reviewing, and editing; EMD: conceptualization, methodology, reviewing, and editing; RW: reviewing and editing; MES: reviewing and editing; CFMF: conceptualization, methodology, formal analysis, reviewing, and editing; SJLB: reviewing and editing; AWG-N: reviewing and editing; GN: reviewing and editing; SPB: reviewing and editing; AÖ: conceptualization, methodology, reviewing, and editing; and all authors: read and approved the final manuscript.

## Data availability

Data described in the manuscript, code book, and analytic code will be made available upon reasonable request.

## Funding

This study was funded by the Dialysis Center Groningen. The TransplantLines Biobank and Cohort study was supported by grants from Astellas Pharma BV (besloten vennootschap) (project code: TransplantLines Biobank and Cohort study), Chiesi Pharmaceuticals BV (project code: PA-SP/PRJ-2020-9136), and Nederlandse Organisatie voor Wetenschappelijk Onderzoek/Toegepaste en Technische Wetenschappen via a partnership program with DSM-Firmenich Animal Nutrition and Health, The Netherlands (project code: 14939). The project was co-financed by the Dutch Ministry of Economic Affairs and Climate Policy by means of a so-called public-private partnership (PPP)-allowances, made available by the Top Sector Life Sciences and Health to stimulate PPPs (project code: PPP-2019-032 and PPP-2022-015). The funders had no role in the study design, data collection, analysis, reporting, or the decision to submit for publication.

## Conflict of interest

The authors report no conflicts of interest.
