# Supplementary material for: Vitamin C status across the spectrum of chronic kidney disease and healthy controls: a cross-sectional study
Source: Am J Clin Nutr. 2025 Sep 8;122(5):1513–23. doi: 10.1016/j.ajcnut.2025.09.008 (PMC12799429; doi:10.1016/j.ajcnut.2025.09.008)
Supplement: Multimedia component 1 [file mmc1.docx]

**Supplementary material: Vitamin C Status across the Spectrum of Chronic Kidney Disease and Healthy Controls: an Observational Study**

**C.S.E. Doorenbos et al.**

**Supplementary text 1.** Vitamin C measurement details

For final determination of vitamin C in plasma and dialysate, vitamin C was transformed to dehydroascorbic acid and subsequently derivatized to 3-(1,2-hydroxyethyl) furo-[3,4-b] quinoxaline-1-one. Reversed-phase high performance liquid chromatography (HPLC) with fluorescence detections(22) was used for the final determination of plasma vitamin C concentrations (HPLC system: Waters Alliance; Column: RP-300 Brownlee (part number 0711-0059), 220 x 1,6 mm, 7µm; Guard column: Aquapore BU-300 (part number 0711-0061 Brownlee), 30 x 4,6 mm, 7 µm; Mobile phase: 21,8 g KH2PO4 dissolved in 1600 mL water with 400 mL methanol, pH adjusted to 7,8 with 60% KOH; Pump: (isocratic) 1,5 mL/min, 5 minutes runtime; Fluorescence detector: Jasco FP2020, λ(ex) 355 nm; λ(em) 425 nm. Injection volume 50 µL.).

| Table S1. Population characteristics of separate dialysis groups. | | | |
| --- | --- | --- | --- |
|  | CHD (n=41) | NCHD (n=11) | PD (n=10) |
| Vitamin C status: |  |  |  |
| Plasma concentration, µmol/L | 30 [15-41]  (range 5-139) | 30 [22-36]  (range 3-54) | 62 [35-76]  (range 6-227) |
| Deficient (≤10 µmol/L), n (%) | 5 (12) | 1 (9) | 1 (10) |
| Inadequate (>10-35 µmol/L), n(%) | 20 (49) | 7 (64) | 2 (20) |
| Adequate (>35 µmol/L), n% | 16 (39) | 3 (27) | 7 (70) |
|  |  |  |  |
| Characteristics: |  |  |  |
| Female, n (%) | 15 (37) | 2 (18) | 4 (40) |
| Age, years | 65 [53-75] | 51 [45-65] | 69 [56-74] |
| Height, cm | 173 [166-182] | 179 [173-182] | 174 [160-180] |
| Weight, kg | 77 [67-85] | 84 [79-103] | 75 [61-87] |
| BMI, kg/m^2^ | 25.0 [22.4-28.4] | 27.3 [24.4-30.9] | 24.6 [22.2-28.4] |
| Systolic blood pressure, mmHg | 152 [137-163] | 127 [109-138] | 137 [121-146] |
| Diastolic blood pressure, mmHg | 71 [60-83] | 67 [55-79] | 69 [65-80] |
| eGFR mL/min/1.73 m^2^ | N/A | N/A | N/A |
| Urine volume, L | 0.4 [0.0-0.9] | 0.0 [0.0-0.0] | 0.7 [0.4-1.5] |
|  |  |  |  |
| Comorbidities: |  |  |  |
| Cardiovascular disease history, n (%) | 22 (54) | 4 (36) | 5 (50) |
| Hypertension, n (%) | 25 (61) | 7 (64) | 5 (50) |
| Diabetes mellitus, n (%) | 13 (32) | 5 (46) | 10 (100) |
|  |  |  |  |
| Medication: |  |  |  |
| Diuretics, n (%) | 21 (51) | 2 (18) | 8 (80) |
| RAAS inhibition, n (%) | 10 (24) | 1 (9) | 3 (30) |
| Betablocker, n (%) | 25 (61) | 5 (46) | 6 (60) |
| Calcium antagonist, n (%) | 17 (42) | 4 (36) | 4 (40) |
| Cholesterol lowering agent, n (%) | 18 (44) | 5 (46) | 2 (20) |
| Oral anti-diabetics, n (%) | 0 (0) | 0 (0) | 1 (10) |
| Insulin, n (%) | 8 (20) | 4 (36) | 0 (0) |
| Corticosteroids, n (%) | 10 (24) | 4 (36) | 0 (0) |
| Calcineurin inhibitor, n (%) | 2 (5) | 0 (0) | 0 (0) |
| Acetylsalicylic acid, n (%) | 19 (46) | 9 (82) | 2 (20) |
| Proton pump inhibitor, n (%) | 24 (59) | 5 (46) | 4 (40) |
| H_2_-antagonist, n (%) | 8 (20) | 2 (18) | 2 (20) |
| Iron suppletion, n (%) | 36 (88) | 9 (82) | 4 (40) |
| ESA, n (%) | 38 (93) | 7 (64) | 9 (90) |
|  |  |  |  |
| Laboratory measurements: |  |  |  |
| Hemoglobin, mmol/L | 7.0 [6.3-7.6] | 7.2 [6.8-7.4] | 6.8 [6.4-7.3] |
| Creatinine, mmol/L | 805 [679-1006] | 749 [621-989] | 895 [805-948] |
| Albumin, g/L | 40 [36-43] | 41 [40-43] | 39 [35-42] |
| CRP, mg/L | 4.1 [1.4-6.3] | 4.7 [3.2-15] | 4.6 [1.0-8.8] |
| Iron, µmol/L | 8.9 [7.7-13.8] | 7.8 [7.0-18.8] | 13.7 [10.9-16.3] |
| Ferritin, µg/L | 404 [198-552] | 293 [148-458] | 251 [179-359] |
| Transferrin saturation, % | 22.5 [13.3-42.8] | 18.0 [14.0-28.0] | 25.5 [17.8-30.0] |
| PTH, pmol/L | 24.6 [13.1-36.8] | 41.2 [11.2-96.8] | 24.8 [8.7-41.5] |
| Plasma sodium, mmol/L | 138 [137-140] | 136 [134-139] | 137 [135-139] |
| Plasma potassium, mmol/L | 5.2 [4.7-5.6] | 4.7 [4.4-5.4] | 4.2 [3.7-5.5] |
| Abbreviations: BMI, body mass index; CHD, conventional hemodialysis; CKD, chronic kidney disease; CRP, C-reactive protein; eGFR, estimated glomerular filtration rate; KTR, kidney transplant recipients; N/A, not applicable; NCHD, nocturnal in-center hemodialysis; PD, peritoneal dialysis; PTH, parathyroid hormone; RAAS, renin angiotensin aldosterone system.  Missing values: CRP 7 in CHD; iron 14 in CHD; transferrin saturation 31 in CHD. | | | |

| Table S2. Dietary intake of separate dialysis groups. | | | |
| --- | --- | --- | --- |
|  | CHD (n=41) | NCHD (n=11) | PD (n=10) |
| Dietary vitamin C intake, mg | 46.0 [24.0-98.5] | 64.0 [14.0-137.0] | 55.5 [25.0-65.3] |
| Dietary vitamin C intake inadequacy (≤75 mg/day), n (%) | 28 (68) | 6 (55) | 9 (90) |
| Prescribed vitamin C supplementation, mg | 35 [35-35] | 289 [289-289] | 83 [83-83] |
| Total vitamin C intake (dietary + supplementary), mg | 81 [58-135] | 353 [303-426] | 138 [107-148] |
| Total vitamin C intake inadequacy (≤75 mg/day), n (%) | 16 (39) | 0 (0) | 0 (0) |
|  |  |  |  |
| Energy intake, kcal | 1539 [1160-2079] | 1656 [1236-1867] | 1648 [1452-1843] |
| Carbohydrate intake, g | 170 [135-215] | 156 [123-221] | 165 [144-186] |
| Protein intake, g | 62.1 [45.6-77.4] | 67.8 [57.0-90.4] | 76.8 [65.5-87.2] |
| Fat intake, g | 61.5 [38.6-92.7] | 68.0 [52.0-82.0] | 67.5 [60.3-90.5] |
| Sodium intake, mg | 1635 [1009-2014] | 1962 [1206-2218] | 1870 [1077-2591] |
| Potassium intake, mg | 2093 [1538-2640] | 2275 [1966-2859] | 2165 [1782-2456] |
| Potassium restriction, n (%) | 20 (49) | 6 (55) | 2 (20) |
| Abbreviations: CHD, conventional hemodialysis; NCHD, nocturnal in-center hemodialysis; PD, peritoneal dialysis.  Missing values: none. | | | |

| Table S3. Vitamin C removal by hemodialysis in separate dialysis groups, using different calculations for estimated ECV. | | | |
| --- | --- | --- | --- |
|  | CHD (n=40) | NCHD (n=7) | p-value |
| Vitamin C clearance (mL/min) | 87.1 [64.1-106.6] | 68.4 [63.0-77.6] | 0.08 |
| Total vitamin C removal (mg) | 58 [27-127] | 128 [97-156] | 0.03 |
| Total vitamin C removal (µmol) | 331 [154-723] | 729 [551-886] | 0.03 |
|  |  |  |  |
| ECV according to Moore et al. (1963) |  |  |  |
| Estimated ECV (pre-dialysis) (L) | 16.7 [15.9-17.9] | 18.6 [17.4-20.1] | 0.16 |
| Total vitamin C in ECV (pre-dialysis) (µmol) | 486 [277-693] | 623 [481-655] | 0.36 |
| Vitamin C removal fraction from ECV | 0.80 [0.57-0.92] | 1.26 [1.12-1.31] | <0.001 |
|  | |  |  |
| Sensitivity analyses: | |  |  |
| ECV according to Moore et al. (1963) + ultrafiltration volume | |  |  |
| Estimated ECV (pre-dialysis) (L) | 19.2 [18.2-20.5] | 21.1 [20.3-23.2] | 0.045 |
| Total vitamin C in ECV (pre-dialysis) (µmol) | 544 [317-772] | 713 [571-756] | 0.34 |
| Vitamin C removal fraction from ECV | 0.70 [0.49-0.86] | 1.04 [0.93-1.16] | <0.001 |
| ECV according to Brøchner-Mortensen et al. (1982) based on weight | | |  |
| Estimated ECV (pre-dialysis) (L) | 13.5 [12.9-14.2] | 14.7 [14.0-15.7] | 0.15 |
| Total vitamin C in ECV (pre-dialysis) (µmol) | 388 [223-550] | 492 [387-514] | 0.38 |
| Vitamin C removal fraction from ECV | 0.99 [0.71-1.17] | 1.54 [1.37-1.63] | <0.001 |
| ECV according to Brøchner-Mortensen et al. (1982) based on weight + ultrafiltration volume | | | |
| Estimated ECV (pre-dialysis) (L) | 16.0 [15.0-16.9] | 17.2 [16.8-19.0] | 0.08 |
| Total vitamin C in ECV (pre-dialysis) (µmol) | 448 [262-632] | 591 [465-623] | 0.32 |
| Vitamin C removal fraction from ECV | 0.85 [0.60-1.05] | 1.20 [1.09-1.43] | <0.001 |
| ECV according to Brøchner-Mortensen et al. (1982) based on BSA | |  |  |
| Estimated ECV (pre-dialysis) (L) | 13.4 [12.5-14.2] | 14.4 [13.3-15.4] | 0.33 |
| Total vitamin C in ECV (pre-dialysis) (µmol) | 401 [220-571] | 475 [378-491] | 0.45 |
| Vitamin C removal fraction from ECV | 1.01 [0.73-1.15] | 1.60 [1.46-1.71] | <0.001 |
| ECV according to Brøchner-Mortensen et al. (1982) based on BSA + ultrafiltration volume | | | |
| Estimated ECV (pre-dialysis) (L) | 15.8 [14.7-17.0] | 16.9 [16.1-19.0] | 0.25 |
| Total vitamin C in ECV (pre-dialysis) (µmol) | 431 [255-648] | 557 [455-610] | 0.32 |
| Vitamin C removal fraction from ECV | 0.83 [0.62-1.05] | 1.26 [1.13-1.46] | <0.001 |
| Abbreviations: BSA, body surface area; CHD, conventional hemodialysis; ECV, extracellular volume; NCHD, nocturnal in-center hemodialysis.  Missing values: none. | | | |

| Table S4. Results of univariable and multivariable linear regression analyses in the CKD population (dialysis group, CKD stage 4/5 group and KTR group combined), with medication use as the independent variable and log2 transformed plasma vitamin C concentrations as the dependent variable. | | | | |
| --- | --- | --- | --- | --- |
|  | Univariable |  | Model 1^1^ |  |
|  | St. β (95% CI) | p-value | St. β (95% CI) | p-value |
| Diuretics | 0.07 (-0.26, 0.40) | 0.69 | 0.14 (-0.20, 0.48) | 0.41 |
| RAAS inhibition | -0.19 (-0.54, 0.16) | 0.28 | -0.02 (-0.38, 0.34) | 0.91 |
| Betablocker | -0.14 (-0.48, 0.19) | 0.40 | -0.04 (-0.38, 0.30) | 0.82 |
| Calcium antagonist | -0.03 (-0.36, 0.30) | 0.86 | 0.08 (-0.27, 0.43) | 0.66 |
| Cholesterol lowering agent | -0.31 (-0.64, 0.01) | 0.06 | -0.31 (-0.64, 0.03) | 0.07 |
| Oral anti-diabetics | 0.10 (-0.55, 0.75) | 0.77 | -0.08 (-0.76, 0.61) | 0.83 |
| Insulin | -0.33 (-0.75, 0.10) | 0.13 | -0.31 (-0.75, 0.12) | 0.15 |
| Corticosteroids | 0.10 (-0.23, 0.44) | 0.55 | 0.27 (-0.33, 0.86) | 0.38 |
| Calcineurin inhibitor | 0.20 (-0.21, 0.61) | 0.33 | 0.28 (-0.27, 0.82) | 0.31 |
| Acetylsalicylic acid | -0.42 (-0.76, -0.09) | 0.01 | -0.47 (-0.81, -0.14) | 0.006 |
| Proton pump inhibitor | 0.09 (-0.24, 0.43) | 0.58 | 0.00 (-0.36, 0.37) | 0.98 |
| H_2_-antagonist | -0.47 (-0.96, 0.01) | 0.055 | -0.39 (-0.93, 0.15) | 0.15 |
| Iron suppletion | -0.08 (-0.41, 0.25) | 0.64 | -0.04 (-0.45, 0.37) | 0.85 |
| ESA | 0.06 (-0.26, 0.39) | 0.70 | 0.00 (-0.50, 0.49) | 0.99 |
| ^1^Model 1: adjustment for age, sex, CRP, vitamin C intake, and study group.  Abbreviations: CRP, C reactive protein; ESA, erythropoiesis stimulating agent; RAAS, renin angiotensin aldosterone system. Missing values: none. | | | | |

| Table S5. Results of univariable and multivariable linear regression analyses in the healthy population (KD group and the HC group combined), with medication use as the independent variable and plasma vitamin C concentration as the dependent variable. | | | | |
| --- | --- | --- | --- | --- |
|  | Univariable |  | Model 1^1^ |  |
|  | St. β (95% CI) | p-value | St. β (95% CI) | p-value |
| Diuretics | -0.15 (-0.44, 0.15) | 0.32 | -0.17 (-0.42, 0.07) | 0.17 |
| RAAS inhibition | -0.11 (-0.41, 0.20) | 0.50 | -0.08 (-0.34, 0.18) | 0.55 |
| Betablocker | -0.20 (-0.55, 0.16) | 0.28 | -0.27 (-0.57, 0.02) | 0.07 |
| Calcium antagonist | -0.06 (-0.42, 0.30) | 0.75 | -0.02 (-0.32, 0.28) | 0.90 |
| Cholesterol lowering agent | -0.17 (-0.45, 0.11) | 0.23 | -0.05 (-0.29, 0.19) | 0.71 |
| Acetylsalicylic acid | -0.24 (-0.64, 0.17) | 0.26 | -0.09 (-0.43, 0.25) | 0.61 |
| Proton pump inhibitor | -0.24 (-0.50, 0.01) | 0.058 | -0.21 (-0.42, 0.00) | 0.055 |
| ^1^Model 1: adjustment for age, sex, CRP, BMI, smoking and eGFR.  Abbreviations: RAAS, renin angiotensin aldosterone system.  Missing values: medication, 25 in KD, 18 in HC; smoking 39 in KD, 45 in HC (711 complete cases in multivariable analyses). | | | | |


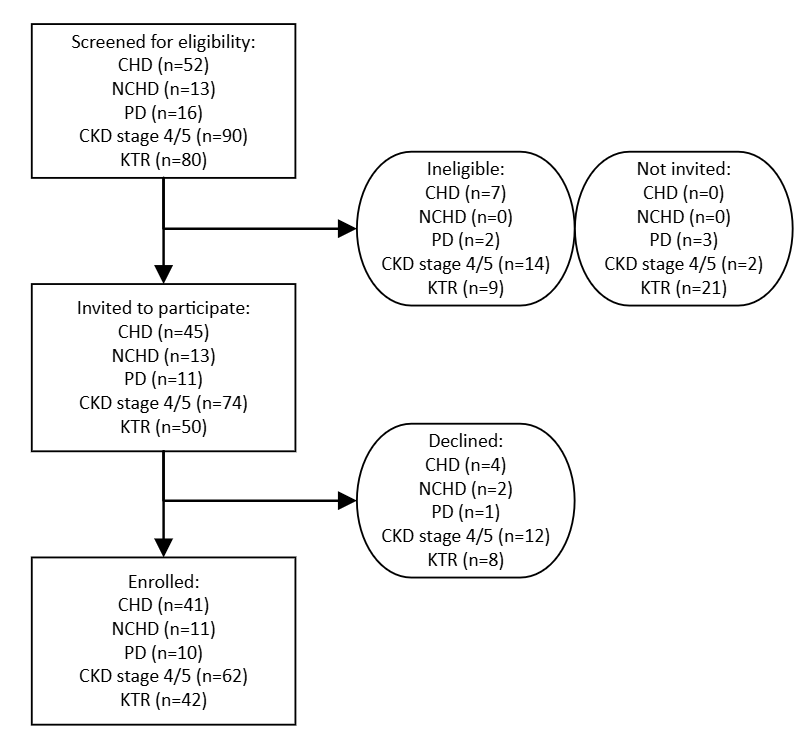


Figure S 1 CKD population participant flow in the study.


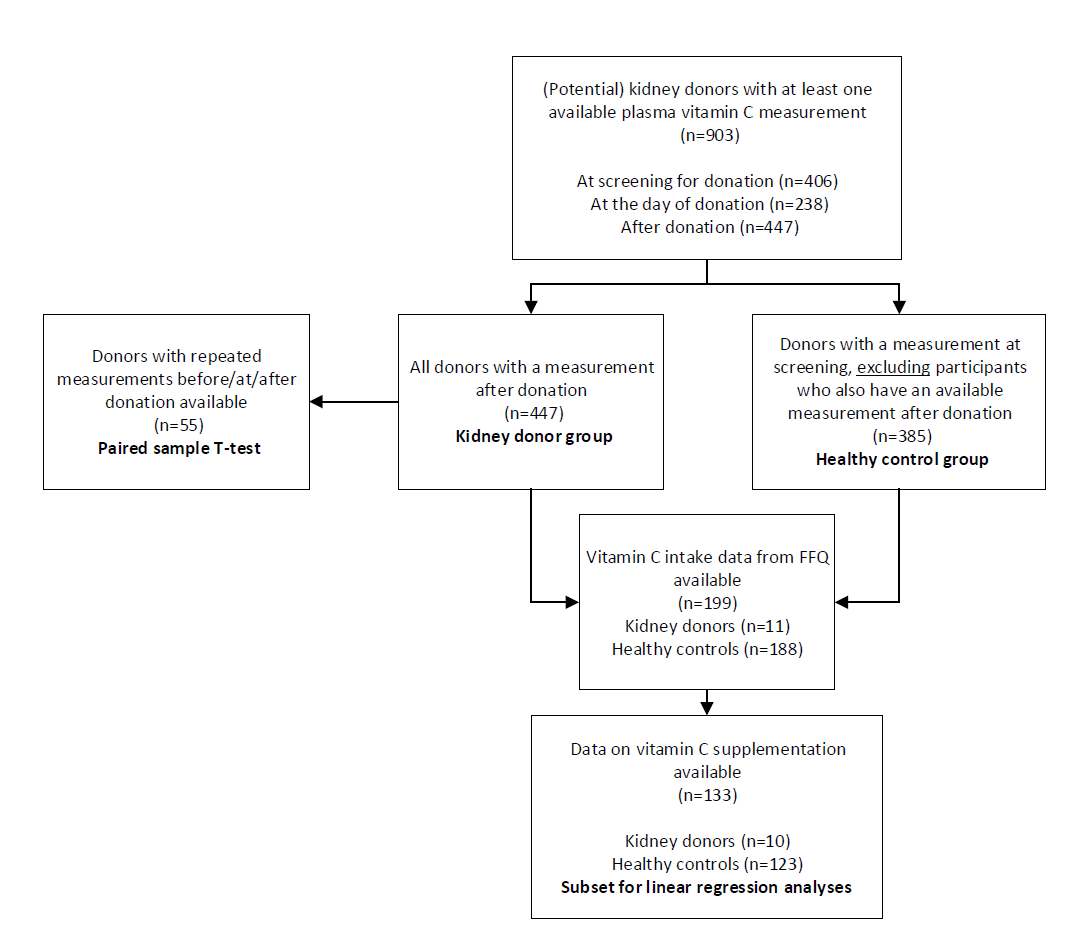


Figure S 2 Healthy population participant flow in the study.
